# Carry-over non-consumptive effects: impact of parasite exposure during larval stage on adult phenotype

**DOI:** 10.1017/S0031182025000265

**Published:** 2025-03

**Authors:** Caroline Liang, Lien T. Luong

**Affiliations:** Department of Biological Sciences, University of Alberta, Edmonton, AB, Canada

**Keywords:** *Drosophila nigrospiracula*, ecology of fear, ectoparasitic mite, interstadial, *Macrocheles subbadius*, NCE, parasitism

## Abstract

The presence of parasites can elicit host responses even in the absence of infection. These risk-induced trait responses include altered host behaviours, morphology, and/or physiology, which can trade off with other fitness-related traits. Studies of predator-induced non-consumptive effects (NCEs) have demonstrated that exposure at one life stage can lead to NCEs in the next stage, but no studies to date have examined such an effect of parasite exposure. Numerous NCEs have been demonstrated in larval, pupal and adult stages of *Drosophila nigrospiracula* exposed to ectoparasitic mites (*Macrocheles subbadius*). Here we experimentally investigated whether parasite-induced NCEs carry over into subsequent developmental stages (i.e. interstadial effects). We tested the prediction that when flies are exposed to mites during the larval and pupal stages, the subsequent adult stage will exhibit decreased body mass, fecundity and longevity. However, we did not detect downstream effects of parasite exposure on adult body mass, fecundity or longevity. The probability of survival and lifetime fecundity were comparable for previously exposed and unexposed groups. We suggest that when parasite exposure is confined to one developmental stage, and the risk of infection is removed in the subsequent stage, the long-term effects of parasite exposure dissipate. The potential to recover from the interim costs of parasite exposure may provide an added benefit to host dispersal.

## Graphical abstract

**Figure:**
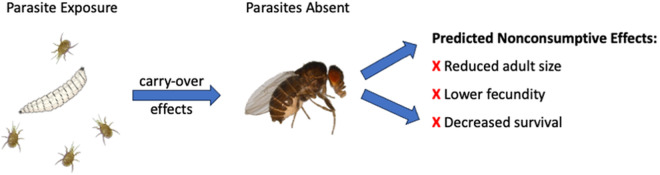

## Introduction

Predators can reduce prey populations by killing and consuming individual prey, but the mere presence of predators can also adversely impact prey populations via non-consumptive effects (NCEs) (Peacor and Werner, 2008; Raffel et al., 2008; Rohr et al., 2008; Clinchy et al., 2013; Peacor et al., 2020). The presence of predators can lead to changes in prey morphology, physiology, life history and behaviour. These risk-induced trait responses can help avoid predation but may also carry costs that trade-off with prey reproduction, survival and density – resulting in NCEs (Preisser et al., 2005; Creel and Christianson, 2008; Peacor and Werner, 2008; Preisser and Bolnick, 2008; Peacor et al., 2020). For example, prey species may reduce activity, change the timing and location of foraging, and shift diet types to reduce predation risk, but these responses may come at the cost of impaired development and growth (Lima, 1998; Hawlena and Pérez-Mellado, 2009; Hawlena and Schmitz, 2010a; 2010b). Given that the threat of predation can be chronic and long-lasting, the ‘ecology of fear’ may have knock-on effects beyond direct predation alone (Nelson et al., 2004; Pangle et al., 2007; Clinchy et al., 2013). Furthermore, predators can exert NCEs on multiple life-history stages of their prey, even on developmental stages that are not typically consumed by the prey (e.g. larval stages of invertebrates) (Ellrich et al., 2016). Short-term exposure of larval *Drosophila melanogaster* to the predator *Phidippus apacheanus* resulted in accelerated development, faster climbing speeds and increased adult survival in the presence of the predator, at the expense of lower adult body mass (Krams et al., 2016). Exposure to predator cues alone during the larval stage can reduce developmental stability and survivorship to the adult stage, and lead to accelerated eclosion, resulting in reduced adult body size, and fewer and smaller offspring (Peckarsky et al., 1993; Stoks, 2001; Elliott et al., 2016; Ower and Juliano, 2019).

A growing number of studies show that parasites, like predators, also exert risk-induced trait responses and NCEs on potential hosts (Raffel et al., 2008; Rohr et al., 2008; Zhukovskaya et al., 2013; Horn and Luong, 2018, 2019), and in some cases organisms avoid parasite and predator cues to the same extent, though in a diametrically opposing manner in some cases (Rohr et al., 2008; C. MacLeod and Luong, 2024). Since parasite exposure can be chronic, organisms may show stronger cumulative responses to parasites than to predators, which are typically more sporadic (Rohr et al., 2008; Buck et al., 2018). The presence of parasites can induce parasite-avoidance behaviours, which are time and energy intensive but necessary to mitigate infection (Sears et al., 2013; Zhukovskaya et al., 2013; Buck et al., 2018; Horn and Luong, 2019; Daversa et al., 2021). Risk-induced trait responses include avoidance of parasite cues (Rohr et al., 2008), reduced activity (Selbach et al., 2022), reduced feeding (Barber et al., 2000; Behringer et al., 2018; Selbach et al., 2022), avoidance of infected conspecifics and changes in habitat use (Hutchings et al., 2002, 2007; Rohr et al., 2008; Fritzsche and Allan, 2012; Daversa et al., 2018). These trait (i.e. fear) responses to parasites, even without direct contact come at a fitness cost (Horn and Luong, 2018). As with predators, parasite-induced NCEs may carry over between developmental stages (i.e. interstadial) effects, even when non-susceptible stages of the host are exposed.

A Sonoran Desert (Arizona, USA) host-parasite association is particularly tractable for investigating the ecology of fear and parasite-induced NCEs. The cactophilic fly *Drosophila nigrospiracula* (Diptera: Drosophilidae) feeds and reproduces on necrotic saguaro cactus (*Carnegiea gigantea*) (Fellows and Heed, 1972; Markow, 1988; Danielson et al., 1994) and is naturally associated with the ectoparasitic mite, *Macrocheles subbadius* (Mesostigmata: Macrochelidae) (Polak and Markow, 1995). Egg, larval, pupal and adult stages of *D. nigrospiracula* can be found on the necrotic tissue of the cactus where mites reside, as such every life stage is potentially in contact with mites. However, only adult flies have been shown to harbour mites, which causes a reduction in fecundity and survival (Polak, 1996). Adult flies court and mate on the exterior surface of necrotic saguaro cactus while females oviposit on newly necrotic tissue where larvae undergo development (Markow, 1988). Female flies avoid oviposition in mite-laden areas and larvae prefer pupating on mite-free substrate; but larvae that are exposed to parasite cues (caged mites) experience lower pupation success (Mierzejewski et al., 2019; Horn et al., 2023). Even though the larval stage is not susceptible to infection, exposure to mites still resulted in a risk-induced trait responses and NCEs on fly larvae. Likewise, pupal stages exposed to mites experienced lower rates of eclosion (L. MacLeod and Luong, 2024).

Although there is increasing evidence that parasite exposure can impact different life stages of the host, little is known about whether parasite-induced NCEs at one stage can carry-over into the next life stage, especially if exposure and hence infection risk does not persist. Here, we investigate the carry-over or interstadial effects of mite exposure by identifying differences in adult phenotype brought on by exposure to parasites as larvae. *Drosophila* larvae display olfactory learning and can avoid parasitoids by detecting their semiochemicals (Scherer 2003; Ebrahim et al., 2015). Pea aphids exposed to parasitoids exhibited increased escape behaviours and consequently reduced feeding (Fill et al., 2012). Likewise, the presence of mites can negatively affect larval feeding and/or pupal metamorphosis with downstream effects on adult fitness traits (Horn et al., 2023). All else being equal, *Drosophila* larval growth and size determines adult body size, and female body size is a significant predictor of fecundity (Lefranc and Bundgaard, 2000). We hypothesized that exposure to parasite cues during the larval/pupal stage imposes NCEs that carry-over into the adult stage, specifically in terms of decreased body mass, survival and fecundity.

## Materials and methods

### Study system

*Drosophila nigrospiracula* (Diptera: Drosophilidae) were collected from necrotic saguaro cacti (*Carnegiea gigantea*) in the Sonoran Desert (Arizona, USA) and used to establish laboratory cultures. Flies were reared in 200 mL bottles in media consisting of instant mashed potato flakes, *Drosophila* medium (Formula 4–24 Instant *Drosophila* Medium, Carolina Biological Supply Company, Burlington, NC, USA), nutritional yeast, and 6 g of autoclaved necrotic saguaro cactus. All fly cultures and experimental flies and larvae were maintained in an incubator (Percival Scientific, Perry, IA, USA) at 24 °C and 70% relative humidity (RH) with a 12L:12D cycle. *Macrocheles subbadius* (Acari: Macrochelidae) were collected from infected flies in the Sonoran Desert and used to establish laboratory cultures. Mites were reared in a 2:1 mix of wheat bran and aspen wood shavings, with free-living nematodes and nutritional yeast. Mite cultures were kept separate from fly cultures in a separate incubator (Percival Scientific, Perry, IA, USA) at 25 °C and 70% RH on a 12:12L:D light cycle. Female mites were extracted for experiments using a Berlese funnel.

### Parasite exposure

In order to obtain a large number of fly larvae for the exposure experiment, larvae were removed with a paintbrush from six culture bottles and transferred to a 50 mL specimen cup of 20% sucrose. The sucrose flotation (Louis et al., 2008) allowed the separation of second and third instar larvae, which tend to float to the solution’s surface (first instar larvae typically sink to the bottom after 20 min). Larval instar was identified according to Bainbridge and Bownes (1981). A total of 50 second instar (L2) larvae were transferred into a single vial, repeated over five control and five treatment vials. These larval vials served mainly to concurrently expose a large larval population to mites, generating sufficient numbers of viable adult females to measure the carry-over effects on fecundity and longevity. Vials (100 mm in length by 20 mm in diameter) contained fresh cactus media (0.9 g instant mashed potato flakes, 0.25 g *Drosophila* medium, 1 g autoclaved necrotic saguaro cactus and 5 mL of distilled water). Treatment vials contained five female mites that were enclosed in a mite cage (2 mL microcentrifuge tube, 40 mm in length by 10 mm in diameter). Both ends of the tube were cropped and sealed with mesh (80 μm pore size) to allow airflow and detection of the mite cues without direct contact with the larvae. Control vials had an empty mite cage. The mite cages were suspended 2.5 cm from the top of the vial using cotton twine rope (50 mm in length by 1.58 mm in diameter) to prevent obstruction of the mite cage by the fly media and to standardize the height at which mite cues were diffusing. Once the larvae reached the third instar (∼3 days), pupation sites were added to improve pupation success. Developing larvae and pupae were exposed to mites for a total of 7 days; mites in the treatment cages were replaced midway through the trial to ensure viability.

### Fecundity and longevity

Upon eclosion, adult flies were sexed, counted and weighed (±0.005 mg, Mettler Toledo XP105, Columbus, OH), and maintained in agar vials. On day 7 a subset of control (5 from each vial, *n* = 25 total) and treatment (5 from each vial, *n* = 25 total) flies were randomly selected to measure survival and lifetime fecundity. A single female fly from the agar vials was re-weighed and moved into a mating vial with two 7-day old unmated males (from the stock culture) and fresh media. Flies were transferred into new vials every 3 or 4 days to reduce larval competition. Males were replaced once a week with 14 day-old males to reduce the effects of age on male courtship and to minimize sperm limitation (Polak and Starmer, 1998). Longevity of the females was recorded with every vial transfer (every 3–4 days). Vials were monitored for eclosion and F_1_ adult flies were removed as they emerged, counted and sexed.

### Statistical analyses

All analyses were performed using the R Statistical Program (R Core Team 2022). Mixed effect models (lmer function, lme4 package) were used to test whether larval mite exposure, fly sex and their interaction affected adult body mass; larval exposure vial and date of emergence (block) were treated as random effects. Proportion of eclosion among the parental generation was not formally analysed since only five replicated vials were initially set up for each exposure condition. Offspring (F_1_) count was analysed with negative binomial generalized linear mixed models where the independent variables included mite exposure treatment, maternal body mass, maternal lifespan and random variables included the maternal date of emergence (block) and the fly identifier (GLMM, lme4 package, MASS package). Survivorship was analysed with the Kaplan–Meier proportional hazard regression model (survfit function, survival package). The Survdiff function (R, survival package) was used to compare survivorship curves between control and exposed flies. Net fecundity of females (L_x_M_x_: average per vial offspring production, weighted by the probability of surviving to that age class) was log-transformed, and a generalized linear model (family = Gaussian) was used to test whether larval mite exposure, fly age and their interaction were predictors of net fecundity. There was a total of 12 vial transfers (every 3–4 days), so each serial transfer approximated a unit of time (3–4 days); this measure of longevity was used for the life table and net fecundity calculations. Stepwise model selection was performed on the models for female body mass, longevity, net fecundity, offspring count. Factors were removed from the models by least significance, and models were compared using the ANOVA function (*χ*^2^, *α* = 0.05), and the change in deviance or the *Χ*^2^ value, and corresponding *P*-value were reported.

## Results

### Adult body mass at eclosion

There was no difference in eclosion rate between control (24.8 emerged adults ± 2.28) (mean ± SD) and exposed larvae (24.0 emerged adults ± 3.39) (mean ± SD). The effect of treatment on body mass was not significant (*χ*^2^ = 1.80, *p* = 0.18). Control (unexposed to mites as larvae) females weighed on average 2.15 ± 0.40 mg (mean ± SD), and control males were on average 1.79 ± 0.27 mg (mean ± SD). Adult female flies that were exposed to mites as larvae were on average 1.97 ± 0.34 mg and exposed male flies were 1.69 ± 0.27 mg. As expected, sex was a significant predictor of body mass at eclosion (*χ*^2^ = 78.32, *p* < 0.001); females (2.07 mg ± 0.382 _S.D._) were 19.0% heavier than males (1.74 mg ± 0.273 _S.D._). The interaction between sex and treatment were significant predictors of body mass (*χ*^2^ = 0.27 *p* = 0.60).

### Offspring count

We examined the effects of mite exposure (during the larval stage) on adult fecundity (in terms of total offspring count). Mite exposure was not a significant predictor (*χ*^2^ = 0.06, *p* = 0.80) of offspring count: control flies produced on average 354.4 ± 43.2 (mean ± SD) offspring and exposed flies produced 329.9 ± 41.6 (mean ± SD) offspring. Lifespan was a significant predictor of offspring count (*χ*^2^ = 40.521, *p* < 0.001). As the lifespan of flies increased, their reproductive output increased correspondingly, regardless of previous mite exposure. There was also a significant positive effect of maternal body mass (*χ*^2^ = 7.71, *p* = 0.006) on offspring count.

### Longevity

**Figure 1.**
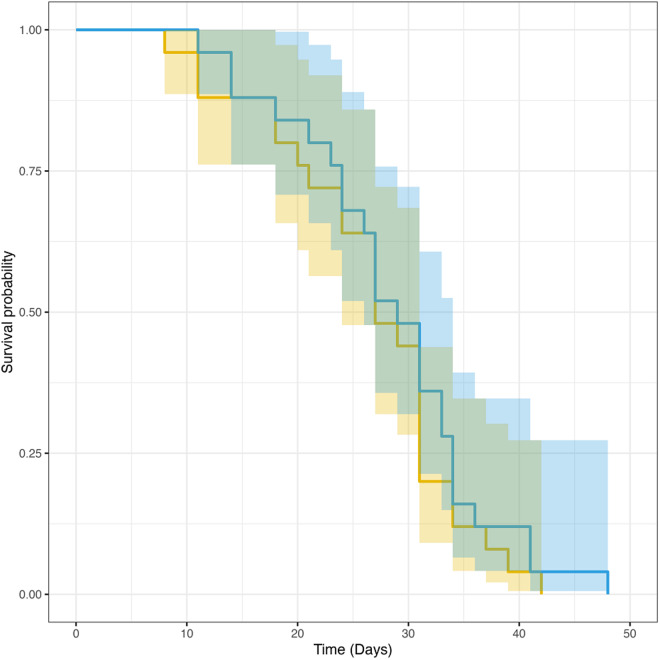
Survivorship curves for flies that were either exposed to mites as larvae (*n* = 25, blue line) or not exposed (*n* = 25, yellow line). The survivorship curves are not significantly different (Survdiff, *χ*^2^ = 0.5, *p* = 0.5). Shaded regions represent 95% confidence interval.

Mite exposure was not a significant predictor of lifespan: exposed flies had on average a 7% increase in longevity compared to control groups, though that difference was not significant (*χ*^2^ = 0.04, *p* = 0.84). Control flies survived 26.6 ± 8.66 (mean ± SD) days and exposed flies survived 28.5 ± 8.84 (mean ± SD) days post-eclosion. The survival curves of control and exposed flies were not significantly different (Survdiff, *χ*^2^ = 0.5, *p* = 0.5) (Figure 1). Mite presence did not affect longevity and mite-exposed flies did not appear to experience an early die off.

### Net fecundity

**Figure 2.**
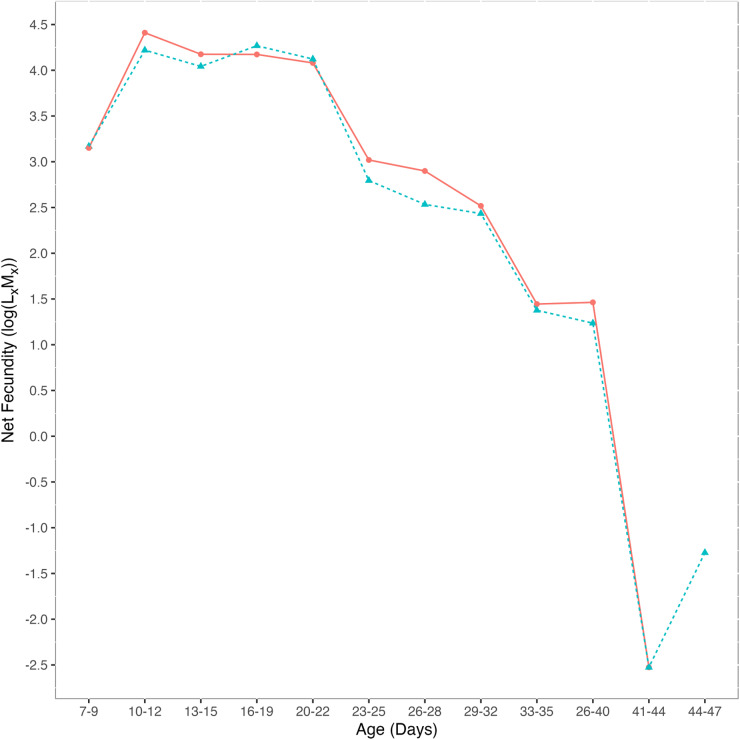
Net female fecundity (log(L_x_M_x_) across age classes). Control flies (red circles, *n* = 25) had no history of mite exposure and mite-exposed flies (blue triangles, *n* = 25) were exposed to five female mites as larvae. There was no statistical difference in net fecundity between treatments (deviance = −0.160, *p* = 0.738).

To account for the effects of longevity on offspring count, we calculated net fecundity (L_x_M_x_). Maternal age class was a significant predictor of net fecundity (deviance = −64.1, *p* < 0.05): net fecundity decreased with age as the probability of surviving to that age decreased. Exposure treatment was not a significant predictor of net fecundity (deviance = −0.160, *p* = 0.74) (Figure 2): there was no difference in net fecundity between exposed and control females. Net fecundity among control flies decreased with age and peaked at 10–12 days post-eclosion (log(L_x_M_x_) = 4.41). Net fecundity among females exposed to mites as larvae peaked later than control flies at 16–19 days post-eclosion (log(L_x_M_x_) = 4.26). However, there was no significant difference in the age at which flies peaked in fecundity since the interaction between treatment and age was not significant (deviance = −0.102, *p* = 0.80).

## Discussion

We tested the hypothesis that exposure to parasites (i.e. in the absence of infection) leads to NCEs that carry-over into the subsequent life stage. Specifically, we investigated whether mite exposure during the larval-pupal stage of *D. nirgrospiracula* impacted adult body mass, reproductive success and longevity. Contrary to our prediction, we did not detect an effect of mite exposure during the larval-pupal stage since there was no difference in adult body mass between control and exposed flies. Only sex was a predictor of body mass, which is expected since female *Drosophila* are generally larger than males (Blanckenhorn et al., 2007; Nunney, 2007). Adult female flies did not suffer reduced fecundity nor reduced lifespan when exposed to mites during the larval-pupal stage. Only maternal body mass and lifespan impacted offspring count, which is as expected since increased insect body size is linked with increased fecundity (Honěk, 1993) and survival (Beukeboom, 2018). The lack of difference in body size may account for the absence of effect of larval exposure on offspring count and net fecundity. Still, these results do not necessarily mean that mite exposure does not have any fitness consequences for flies. Since pupation success was not explicitly measure in this study, we effectively only measured fecundity and longevity for flies that managed to survive to the adult stage. A previous study showed that *D. nigrospiracula* larvae exposed to mites had lower pupation rates and preferentially pupated in mite-free zones (Horn et al., 2023). Similarly, pupae exposed to mites experienced lower rates of emergence into the adult stage (Liang and Luong, 2024).

Another possible explanation for the lack of effect of larval-pupal exposure on adult phenotype pertains to the quality of the larval environment in our experiment. The exposure vials we used were comparably larger with more headspace than the Petri dish arenas used in the study by Horn et al. (2023), which may have resulted in more diffuse mite cues in our study. Mite cages were suspended above the media to prevent the media from clogging the mesh screens, but may also increase the distance between larvae and parasite cues. Also, the current experimental set-up did not offer larvae a choice between mite-free and mite-laden environments for pupation, yet habitat avoidance can mitigate infection risk (Daversa et al., 2018; Horn et al., 2023). Although larvae formed pupae along the length of the vial, previous attempts by L. MacLeod and Luong (2024) to expose pupae to mites only yielded NCEs when mites were in direct contact with the pupae and not through a mesh screen, as was the case in this study. Larvae spend much of their time burrowing into the cactus media as they feed (Fellows and Heed, 1972; Danielson et al., 1994), and this behaviour likely buffers against exposure to parasite cues. Therefore, the media in the vials may have provided transient mite-free refuges that compensated for the adverse effects of mite exposure. Future studies should increase the concentration of mite cues by increasing mite density or minimize refuges. In *D. melanogaster*, predator cues had a greater impact on fecundity and offspring mass at low fly densities (less than 20 individuals per vial) (Elliott et al., 2016, 2017).

The availability of resources may also dampen the deleterious effects of parasite exposure on body size and other fitness traits. The larvae in our study were provided with high quality food *ad libitum*. However under conditions of nutritional stress, greater differences in body mass may arise, which would directly impact longevity and reproduction (Ower and Juliano, 2019). Indeed, female *D. nigrospiracula* infected with *M. subbadius* had higher fecundity with yeast-supplemented diets compared to uninfected females with yeast-free diets (Polak, 1996). Future experiments could test whether nutritional availability interacts with parasite exposure to accelerate development (e.g. to escape the threat of parasitism), but at the cost of growth. Alternatively, it is possible that the NCEs of mite exposure on larvae simply do not affect adult survival and fecundity (once they successfully emerge from the pupae). According to Moran (1994), organisms with distinct developmental stages (e.g. amphibians and invertebrates) are adapted to decouple developmental processes such that each developmental stage can respond independently to different selective pressures. This ‘adaptive-decoupling hypothesis’ could explain the lack carry-over effects in this system. Moreover, if adults disperse to a new habitat with no or few mites present, the lack of any carry-over effects may allow flies to recover from the NCEs experienced in earlier life stages, providing an additional benefit to dispersal. Hence, spatial heterogeneities in parasite risk could influence the expression and magnitude of NCEs over the lifespan of hosts.
